# Aspects of Developmental Pathways toward World-Class Parasport

**DOI:** 10.3390/sports10080123

**Published:** 2022-08-15

**Authors:** Lise Storli, Morten Andreas Aune, Håvard Lorås

**Affiliations:** 1 Department of Sport Science, Sport and Human Movement Science Research Group (SaHMS), Nord University, 7600 Bodø, Norway; 2 Department of Teacher Education, Faculty of Social and Educational Sciences, NTNU—Norwegian University of Science and Technology, 7491 Trondheim, Norway

**Keywords:** para-athletes development, sport expertise, deliberate play, deliberate practice, developmental model of sport participation (DMSP)

## Abstract

The developmental pathways of athletes with a physical disability into world-class parasport are much less researched and understood compared to able-bodied athletes’ participation histories. The purpose of this study was to investigate the developmental pathways of para-athletes toward elite performance. Data from eight athletes with physical disabilities ranked among the top performers in Paralympics, World Championships, and/or European Championships were gathered. Thematic analysis of retrospective semi-structured interviews led to the identification of four themes. The findings showed the importance of early childhood sport-related encounters in a family environment followed by sampling of various organized and coach-led sports throughout the childhood period. The youth sport period was highly heterogenous, albeit with important transitions towards elite-level practice environments, competition, coaching, equipment adaptability and the first intentions of becoming an elite-level athlete. Throughout, significant contributions are attributed towards parents, friends, coaches, athletes, and others, towards fostering a sustained motivational climate focused on improvement and further progress. These findings could provide useful information to tailor developmental models towards elite-level performance in parasport.

## 1. Introduction

The developmental pathways towards expertise and peak performance in sport has been investigated in abundance the past three decades, highlighting the individual and sport-specific interplay between factors such as deliberate practice or play, specialization or diversification, participation in organized vs. unorganized activities, and coach-led vs. self-initiated practice in training and competition [1,2]. In contrast, the developmental pathways of elite-level athletes with a disability have not been subjected to similar empirical investigations in order to disentangle important aspects of their pathways towards elite level performance. Parasport is increasingly becoming recognized as a legitimate component of international sport, although in some instances the elite-level athletes with disabilities are still not taken as seriously as elite-level able-bodied athletes [3].

Parasport is simultaneously used as a synonym for Paralympic sport, which addresses athletes who compete in the Paralympic Games [4]. The term captures practice and competition for persons with a physical or intellectual disability, in which the type of disability determines types of competitive categories in order to ensure fair competition amongst athletes with similar disabilities [5]. To be eligible for parasport, a person must have an impairment type according to the minimum disability criteria outlined in the World Para Athletics Classification Rules and Regulations [6]. Illustrating the substantial individuality and diversification amongst athletes with a disability in general, World Para Athletics describes eight eligible physical impairment types which apply to various competitive events, creating around ~100 different competitive classes. The main aim of the current study was to investigate the developmental pathways towards elite performance (as defined by top-level performance in Paralympics and/or World/European championships) among athletes with a physical disability. As parasport clearly contains a substantial heterogenous sport population due to individual differences related to (amongst other factors) type and degree of disability, the developmental pathways towards elite-level performance might potentially be more diversified in athletes with a disability compared to able-bodied athletes who typically demonstrate at least some degree of homogeneity in physical characteristics according to sport-specific demands. Thus, data on developmental pathways towards elite-level performance obtained from able-bodied athletes might not necessarily be applicable toward elite-level parasport.

Apart from the potential differences in physical characteristics between able-bodied and athletes with a disability that might implicate differences in developmental pathways, young participants in parasport typically have far less opportunities in terms of organized club-sports, local, regional and national developmental/talent programmes/teams, highly experienced and educated coaches, overall funding, as well as other organizational factors that exist for able-bodied athletes [4]. Many nations worldwide still lack appropriate systems from local up to national level that address parasport in general, and particularly when it comes to high-level parasport [7]. Thus, athletes with a disability might already experience substantial barriers towards sport participation (in general) at a local level, which might impact upon their developmental pathways towards elite performance levels.

Existing research on key determinants in development of elite sporting performance amongst athletes with a physical disability is relatively scarce. In a systematic review from 2017, Denghansai et al. [8] noted that few studies had examined issues relating to trajectories, training modifications, competition, or recovery processes throughout the sporting careers of athletes with a disability, from their introduction to sport-related activities through all phases on the path to competing at the highest level of international competition in their particular sport. Some authors have claimed that athletes with a disability pass through the same sport-related developmental stages as able-bodied athletes, only with some modest differences in rate of progress depending on the degree of disability [9]. Results from two studies on elite Canadian wheelchair basketball players, however, indicate that factors such as athletes’ disability classification and acquired vs. congenital disabilities introduce inter-individual variability in terms of reaching performance-related milestones, and impacts upon developmental pathways in training and competition history [10,11,12]. Further, Denghansai et al. [13] compared developmental trajectories between elite able bodies, and para-athletes with both congenital disability and acquired disability, and the results showed both similarities and differences between the three groups, but more interestingly, it was shown that congenital disability Para-athletes had more developmental similarities to able-bodied athletes.

Developmental pathways toward elite-level sport performance, from the first experiences with sport up to top performances in major international tournaments, are typically examined by referring to athlete development models. In the current study, we examine developmental pathways towards elite-level parasport through the lens of the Developmental Model of Sport Participation (DMSP) as a theoretical framework [14]. Albeit caution needs to be exercised in applying a model of athlete development initially designed for able-bodied athletes, the DMSP postulates three broad stages in which high-performance athletes move through on their way to expertise through childhood and adolescence, involving sampling, specialization, and investment. Sport specialization is defined as year-round training greater than 8 months, choosing and focusing on a single primary sport [4], while sampling involves sustained participation in several sports. The key element in sport sampling is that children experience and develop a number of different motor skills and cognitive skills that are crucial to optimize skills in the main sport, and sports specialization is aimed to develop sport-specific motor coordination patterns through a high amount of specific training [1,15].

Thus, the DMSP outlines two possible developmental trajectories towards elite-level in sport: (1) elite performance might emerge from a combination of primary sampling in the childhood years and later specialization in adolescence, or (2) elite-level performance emerges through early and sustained specialization in childhood and adolescence. The DMSP predicts that elite performance through sampling consists of specializing years around the ages of 13–15 succeeded by primary sampling years in childhood. In this pathway, high amounts of deliberate play are suggested as a significant factor towards staying motivated with sustained enjoyment. In comparison, the second developmental trajectory toward elite-level performance in sport contains early specialization and focus on one sport and performing high amounts of sport-specific and individualized coach-led deliberate practice [1]. The past two decades of work associated with the DMSP framework on elite athletes has generated seven postulates that have received sufficient support from research [16,17]. Despite the general consistency of trends leading up to postulates in elite able-bodied athletes, their relevance for understanding the development pathways of elite-level athletes with disabilities is relatively unknown. The first three postulates address early diversification (sampling of various sport-related experiences) as favorable for long-term sport involvement, positive youth development, and development of high-level performance. Regarding athletes with disabilities, they might in principle not have the opportunity to ‘sample’ several sports in childhood due to a lack of organized practice and competitions from local clubs and sport organizations. Taking part in several sports has been suggested to optimize the development of motor skills to athletes’ main sport [15,18], although in regard to parasport some studies could not draw a similar conclusion [19].

Two other postulates originating from the DMSP highlight the importance of high amounts of deliberate play in the sampling years to build strong intrinsic motivation and establish a range of motor and cognitive competencies that children can ultimately bring to a main sport [16]. Deliberate play concerns the involvement in youth-led informal sports-play with peers (such as playing, leisure time, backyard soccer, street hockey, basketball in the driveway, ice-hockey on a frozen lake, etc.) without adult/coach supervision [15,20]. These activities are for the purpose of maximizing enjoyment, freely chosen, and are regulated intrinsically by the children/adolescents themselves where rules are typically adapted from original formal sports. However, athletes with a disability might experience similar barriers for participating in such informal physical activities, as for participation in organized sport. One typical recurring theme when it comes to sport participation for athletes with a disability concerns the important role of individualized equipment [21]. Thus, lack of resources and/or availability of equipment might be just as consequential for athletes with a disability towards participating in deliberate play-related activities as for their entrance and participation in organized sport.

The last two postulates from DMSP address the adolescence period and stress the opportunity for athletes to choose to specialize in their favorite sport in this developmental period [16]. Especially in late adolescence, the physical, cognitive, social, emotional, and motor skills are considered developed at necessary levels for youth athletes to invest effort in highly specialized training in one sport [1,22]. However, this might not necessarily apply to all parasport athletes, because selecting a sport implies that there is a pool of sports to select from. Furthermore, the necessary levels of elite-sport readiness in terms of biological and psychological characteristics might not be similar in athletes with a disability compared to able-bodied athletes.

Based on the presented considerations, the main aim of the current study was to further examine the developmental pathways towards elite-level performance, through childhood and adolescence, amongst athletes with a physical disability that can be defined as experts in their fields. Thus, athletes included in the current study are competing at the highest level and are ranked amongst the top performers in Paralympics, World Championships, and/or European Championships. By conducting retrospective qualitative interviews, the purpose was to examine the developmental histories and experiences of elite athletes with a physical disability, including exploring their perspectives and reflections in terms of the proposed pathways in the DMSP. The goal was to further understand aspects of the various developmental periods and milestones involved in pathways toward world-class performance in parasport, in an attempt to shed light on and address some of the proposed problems with applying non-disability athlete development models such as the DMSP towards understanding athletes with a physical disability. Further understanding of the developmental process by recognizing the experiences of these athletes could potentially inform the training and development of elite parasport.

## 2. Materials and Methods

A strategic sample of eight elite-level world-class athletes with a physical disability were recruited by contacting sport federations and through personal contacts. To capture some of the variations in elite parasport, five male and three female athletes were included, and they were recruited from both winter and summer sports as well as individual or team sports. Furthermore, the sample contained five athletes with acquired disabilities in the lower extremities (progressive neurological disease, amputation, and/or paralysis) and three athletes with congenital disabilities in the lower extremities (paralysis and/or spinal hernia). To be included, they had to compete and perform at the top level in Paralympics, The World Championships, The European Champions, or several of these. In the recruited sample, the average age of the participants at the time of data collection was 27 years (SD = 7.5 years). As a testimony of the elite level of the athletes with a physical disability in the current study, six of them had won medals (1st, 2nd or 3rd place) in one or several of the mentioned competitions, and the two remaining participants had competed in the Paralympics and/or World Championship with results among the top 5. All participants provided written informed consent before completing the interview, and the project was approved by The Norwegian Centre for Research Data. Because of the Norwegian law regulated through the Norwegian Personal Data Act and The General Data Protection Regulation from the EU (GDPR), we are prohibited from providing more personal information about the participants in order to preserve their anonymity. Truly elite-level world-class athletes with a physical disability are not a substantial population, and connecting information through, e.g., reporting gender, type of sport, type of disability, age, etc., together could easily lead to readers potentially identifying a specific participant.

### 2.1. Philosophical Assumptions

The study adopted a qualitative approach as it provides an analytical framework that potentially generates deep insight into parasport athletes lives during their developmental pathway toward world-class performance. Informed by a post-positivistic epistemological and ontological position that stresses the potential bias in considering the researcher as an independent and completely objective observer [23], the authors acknowledged the close connection between researchers and the research participants. The research is thus not conducted on the elite parasport athletes, rather, the research unfolds to learn from the athletes. This calls for a strive to engage in social construction of a narrative regarding the developmental pathway of individual participants, in an attempt to activate the respondent’s knowledge and experiences.

To gain insight into athlete’s developmental pathways toward elite-level performance, there needs to be reflections made by the research team toward how one’s values, beliefs and experiences influence especially the analytical process. The researchers involved in the project had a mixture of experiences from various practical fields such as physical activity, sport, and physical education, with scientific approaches derived from the fields of psychology, human movement science and sport science and with a diversity of research methodology. All members of the team also had experience in working within sport and physical recreation with able-bodied children, adolescents and adults, and the first author has experience with coaching athletes with a disability. When the study was conducted, however, none of the researchers were directly involved with parasport. Thus, the diversification of the research group helped strike a balance between taking a distanced view and being sensitive to contextual features. By acknowledging that valid knowledge claims might emerge through differences in understanding [24], interpretational possibilities of interwoven ideas from issues raised during the interviews, the participants’ reactions, and our interpretations of these, were discussed and negotiated among the members of the research group. Research in this mode thus required an ability to ‘see the whole picture’ while avoiding simply aggregating data in order to arrive at an overall conceptualization [25].

### 2.2. Retrospective Interviews

The athletes participated in an interview based on retrospective recall methodology, designed to examine their sport practice and developmental histories. All interviews were collected individually by telephone and the audio was recorded by a SONY IC recorder ICD-PX370 (Tokyo, Japan). The interview was conducted in the mother tongue of both interviewer and participants and lasted approximately 40 min, and all were conducted by the same interviewer (the first author). The final part of each interview was a member-checking procedure [26], in which participants were provided a summary from the interviewer’s notes and asked to provide input on whether it accurately reflected their developmental experiences.

Retrospective semi-structured interviews are considered as one of the superior approaches to collect historical practice data in sport as they allow for additional probing to obtain as rich information as possible [27]. They provide an opportunity for minimizing misunderstandings, and openings for additional clarification [28]. Thus, they have been used extensively in previous research on athletes’ development toward elite (expert) levels [1,27,29]. In the current study, structured and habitual sport activities were addressed in athletes that are currently engaged in sport participation, which provides for shorter recall periods with salient events, that has been shown to provide accuracy in retrospective recall data [30]. Furthermore, elite athletes also typically produce training dairies at an early age and follow individually tailored training schedules developed by their coaches which enhance memory of previous practice schedules. It should be noted however, that elements of bias necessarily apply to all types of retrospective recall data [30], albeit studies have demonstrated that athletes can reliably report details of physical activity patterns up to 25 years retrospectively [31,32,33].

### 2.3. Structure and Topics of the Semi-Structured Interview

According to the developmental model of sport participation (DMSP), the semi-structured interview was portioned into four main developmental periods: childhood (up to 12 years of age), youth (from age 13 up to 16), junior (age 17–19), and senior level (age 19+). Across all periods, they were asked to reflect upon participation in organized competitive sports (practice and competition), transitions between periods, time spent in organized practice (with a coach) and unorganized self-monitored practice. The organized training was characterized by coach-led training sessions with instructions and feedback while unorganized training was defined as individual or self-led activities [28]. Furthermore, the athletes were also asked to reflect upon the development of relevant equipment (as this is a recurring theme in parasport), training schedules, and their contact with local, regional and national sport federations.

Explicitly for the developmental period defined as childhood, the athletes were asked to recall their first sporting experiences, i.e., when they first tried a sport-related activity (organized or unorganized). In youth, transitions from childhood were considered in terms of changes in type of sport, unorganized/organized activities, competitions, and whether they began reflecting upon becoming a high-performance athlete. In junior age, the transition from primary school to high school was investigated, and how sport participation affected everyday life. According to DMSP, many athletes develop a strong motivation for becoming a world-class athlete in their sport, and they were thus asked to reflect upon important factors for becoming elite performers especially in this period.

### 2.4. Thematic Analysis

Recorded audio was transcribed and analyzed by a six-step thematic analysis [34]. This included a reflective approach in which we systematically and iteratively worked forward and backward through the steps of (I) familiarization with the data, including transcribing and noting down initial reflections, (II) generating initial codes for each participant’s transcripts (across the entire dataset), (III) development and searching for themes across participant’s individual codes, (IV) reviewing themes according to initial codes and the entire dataset, (V) defining and naming themes and (VI) writing up results. As a thematic analysis emerges in the interaction between research data in relation to the researcher’s theoretical assumptions, knowledge, skills, and experience [34], the approach in the current study can be defined as a more explicitly analyst-driven theoretical thematic analysis driven by the current study’s approach rooted in the developmental model of sport participation (DMSP). Albeit this form of thematic analysis might risk a somewhat reduced description of the overall data in exchange for a detailed deductive analysis of some aspect of the data, the data analysis was still data-driven and guided by the above systematic thematic analysis phases advocated by Braun et al. [34,35].

### 2.5. Study Rigor

All three authors were involved in the data analysis and contributed to the analytical rigor. Data were managed using NVivo 11 (QSR International, Chadstone, Australia). Throughout the analysis, actions were undertaken to ensure rigor, including archiving individual data analysis files and decisions from critical discussions [26]. First, the researchers operated in isolation from each other to independently code data without negotiation, before further critical discussions between three authors were undertaken and maintained throughout the six-step thematic analysis. These discussions focused on the importance of exploring how athlete development towards elite levels was understood and described by elite athletes with a physical disability, as opposed to previous studies that focused on the developmental pathways of able-bodied athletes. The discussions were also vital in ensuring and maintaining reflexive examination of assumptions and the authors’ own prior views throughout each stage of data analysis.

## 3. Results

The overarching theme of the current study was the developmental pathways of elite athletes with a physical disability, viewed through the lens of the developmental model of sport participation (DMSP). Although each athlete’s developmental pathway into elite parasport was unique, strong commonalities across their experiences were also apparent and were captured in the following four themes: (a) initiation of sport-related activity, (b) sport sampling in childhood, (c) youth sport transitions, and (d) significant others. The theme names were selected in keeping with the athlete’s descriptions and the DMSP terminology.

### 3.1. Initiation of Sport-Related Activity

A recurrent theme among the interviewed elite athletes with a physical disability, was the relatively early introduction to sport-related activities in their childhood, in which several described experiences already in the preschool age (2–4 years old). This activity was typically in leisure time with their families, and many had vivid memories of these events:

I was 4 years old when I got a ski-sledge…I wanted to ski on my own, so this was one of the best winter seasons [at this age] as I could move around on snow on my own without any help…

Around the age of entering the first grade at school (5–7 years old), all athletes were introduced to what might be defined as organized sport (typically by a local sport club). Many athletes in the current sample report this as an important part of participating on the same level as their peers and looking for sport opportunities rather than seeing the activity limitations of their disability:

It has always been very important for me to do the same as my friends. So, I played multiple sports. It has always been important to see opportunities instead of limitations…

### 3.2. Sport ‘Sampling’ in Childhood

There are several instances in the transcripts that can be defined as sampling according to the developmental model of sport participation. Altogether the athletes report a total of 16 different team- and individual sports, in which none reported sustained activity in less than 3 of these throughout their childhood years (6–12 years old). As one athlete noted:

I tried and practiced handball, athletics, swimming, football, alpine skiing, table tennis, tennis, badminton and sit-skiing. I have tried most sports, although in some it was initially difficult to participate, but with a little adjustment, it went very well!…

Besides the overall multi-sport approach as an indication of sampling, the participation rates also indicate substantial time and involvement in the various sports. All participated in organized sport training several times a week and reported an average rate of 118 yearly practice sessions in childhood, as well as some local club-initiated competitions, in addition to unorganized sports-related training/activity in leisure time typically with their friends. Still, the playful approach is clearly visible across all their activities:

It was mostly training for fun when we were little. It was some organized training with the team, but since we were so small it was not serious…

### 3.3. Youth Sport Transitions

The youth sport period seemed to overall represent the developmental period of greatest diversity among the athletes in terms of their sport practice and competition. This is captured by the many transitional experiences laid out by the participants. First, albeit all report a mixture of organized and unorganized (self-initiated) sport activities and an increase in overall sustained activity levels, some increase their involvement in organized sport while yet others spend more time in self-initiated sport activities before eventually changing back to organized sport:

I practiced with coaches [organized] in soccer and handball, but unorganized on skateboards and snowboards. I also practiced some organized floorball…

All of the elite athletes in the current study also reflected upon their transitions towards more organized and professional training and competition environments. This shift in environment was due to change of schools (sometimes involving moving to another place in country) and/or being approached (or seeking out on their own) organized parasport typically in bigger sport clubs in terms of memberships. Amongst other things, these transitions involved the introduction of training schedules, albeit most of the athletes did not get a professional schedule developed in collaboration with a coach until late adolescence. The initial schedules were typically described as very general and contained very little individual adaptions:

It was a very general training schedule, some physical things such as strength and endurance, but very different training methods I should try out. Getting a feel of the different heart rate zones…

The youth sport period also seems to represent a significant shift towards participation in competition:

Initially, I participated mostly in regional competitions. I had not yet been introduced to parasports, so I competed mostly against the able-bodied. From the time I was 14–15 years old, I was classified as a para-athlete and got to participate in the National Championships and joined the national team for young upcoming athletes.

Thus, many athletes experienced their first transition towards both practicing with, and competing against, other athletes with a disability at a relatively high level. As the transcript indicates, some had their first experiences altogether with parasport competitions in the youth sport period.

Individual adaptations in equipment represent a reoccurring theme in relation to parasport at all levels. In the current study, the participants also mention the role of equipment as a significant developmental factor. As they typically start out with limited access due to expensive (and sometimes non-existent) equipment and may have to rely on custom-made equipment made by their families, the youth sport period is where most experience transitions towards lighter and more individualized equipment. This is especially important as the athletes also pass through puberty with a possibility for substantial variations in growth and maturation that might amplify the need for specially adapted equipment:

The [available] equipment has been fine, however too expensive. …I know several athletes who have had problems getting their own equipment. I went to buy my own equipment because they had not brought that type to my country yet…

A fifth transitional factor occurring in the youth sport period amongst the athletes in the current study concerned a shift towards intentions of becoming an elite performer:

My first international competition was at the age of 14, and I did not perform at a high level. It was around that time I realized that this was something for me if I put in a lot of training…

Thus, the competitive spirit and desire for further improvement typically emerges around their first experiences with high-level sport or around other transitional factors such as changing team/club or sport:

When I moved to a bigger city, there was an opportunity to practice with the very best players. In fact, there were several players so you could play and practice as a team…

### 3.4. Significant Others in a Sport Context

A recurring theme amongst the athletes in the current study, was the role of significant others throughout their developmental pathways, and their contribution toward initiation and sustained involvement and investment in sport. In childhood, parents play a particularly important role:

Parents are important supporters. They do not hold back, instead they push forward for further development…

Parents, coaches, peers, and family are all assigned the crucial role of helping to see beyond the functional limitations associated with various disabilities and focusing on possibilities. In the youth sport transitions especially, training and practicing with qualified and experienced coaches across an extended period of time are highlighted as a key factor towards further development:

I had a good coach who was very experienced and who knew how to push me to get even better… He has followed me all the way from early years and is the national team’s head coach today…

However, coaches had also been situated in clubs with a community and culture of sharing, where athletes show interest in each other’s development:

To get into an environment and training group have been extremely good for me. It is an enormous sharing culture, which has given me access to the world’s best athletes training journals…

Many of the elite athletes in the current study also address the possibility for training and practice with athletes without disabilities:

I have gained a lot in terms of technique and training methods when I have pushed myself with able-bodied athletes. To participate with them, you get into a culture that is very professional, and it does not get much better…

National-level sport federations working with the development of elite athletes both with and without disabilities have an important role in this regard, and are often mentioned as a key for individual development from sub-elite towards elite sport performance:

[the national federation]… has been there from an early age, and we received training schedules and got feedback… when you have such resources it is important to use them…

## 4. Discussion

The findings of the present study offer support for some of the propositions in the developmental model of sport participation (DMSP) applied to elite athletes with a physical disability. Specifically, there seems to be an important role for sampling organized sport in childhood, i.e., trying out and participating in several sports. Also consistent to a degree with the DMSP, is the opportunity for athletes with a disability to choose to specialize in their favorite sport during the period of adolescence [16]. This includes sustained investment and effort and increased specialized training in one sport. The reflections of elite-level athletes with a physical disability on the various developmental periods, however, offer important nuances of the DMSP framework applied to elite parasport and highlight important factors involved in determining the opportunities for pursuing high-performance and elite-level parasport. It needs to be acknowledged that any developmental history of sport participation is in essence highly individualized. As so in the current sample of elite-level athletes with a physical disability, the youth sport period was especially heterogenous, in which the common factor was in principle that it was highly transitional in many aspects.

### 4.1. Initiation of Sport-Related Activity

The DMSP framework initiates with ‘entry into sport’, and further describes either sampling or specialization through sport participation in childhood [14]. There is, however, no substantiation of what constitutes the entry-phase and what might be crucial determinants in terms of getting children into sport or whether some factors might be important in terms of long-term participation towards elite-level performance.

As our study shows, all para-athletes started with sport-related activities at relatively young ages. However, this is in contrast to studies on Brazilian para-athletes with both congenital disabilities and acquired disabilities, where it was shown that para-athletes tend to start with sport in older ages compared with able-bodied athletes, and indicate that early starting age in parasport is not crucial to achieve expert level [12]. Respectively, this underpins that para-athletes can still be successful and maintain long-lasting careers in sport, even though they started with sport in older ages [12]. These, to some extent, conflicting results might be explained since both studies include different respondents and subsequently indicate that starting age is highly individual in parasport. However, a novel aspect of the current study was when the elite athletes were asked to reflect upon their entry into sport, they did not initially talk about their first introduction to organized and coach-led sport in local club, rather, they reflected about their early childhood experiences in leisure time with their families trying out different sport-related activities typically before the age of 5–6 years old. In this period, they had important experiences with physical activities that might have initially ‘shaped’ their personal understanding as a possible sport participant, including first experiences with personalized equipment that allowed them to move around freely during all seasons.

These early family experiences thus seem to be an integrated part of the entry into sport for the elite athletes in the current sample. Indeed, an emerging body of research strongly suggests that socialization in the family and ‘family culture’ is one of the strongest influences on children’s propensities to take up sport [20,36] and can have profound and lasting effects on individuals’ sports participation across the life course [37]. Thus, parents are thought to be the most influential socializing agents for children’s early sport-related learning experiences and certainly the first point of socialization into sport and other leisure activities [38]. The family culture might therefore be especially important for athletes with a disability, as parents in particular can facilitate the introduction of many different sport-related activities and broaden their horizon towards seeing physical activity possibilities, rather than negotiating their disability. This also includes addressing the need for, and application of, individualized equipment as a part of the demonstration of sport opportunities and thus perhaps ‘setting the stage’ for identifying themselves as a possible athlete and sport participant.

As an integrated part of the entry into sport, the current sample of elite athletes with a physical disability also stressed the importance of the desire to do the same as their friends and being allowed to participate with their able-bodied peers. As peer environments may vary considerably because of demographic and socioeconomic factors, athletes with disabilities might be introduced to a vast continuum of social environments. These might range from dedicated peer groups where peers share similar disabilities and even experiences, reverse-integrated or inclusive groups where peers vary regarding ability and may include able-bodied participants, up to being the single minority in an otherwise able-bodied peer group [39,40]. At the time of entry into sport, all elite athletes in the current study experienced the latter scenario, and thus were integrated into an otherwise able-bodied peer group in the local sport club situated in a relatively small community. This clearly illustrates that early on in the developmental pathway towards elite parasport, it might be just as (or perhaps more) important to participate with able-bodied friends and peers in sport, as there is a need for dedicated peer groups/clubs exclusively for young people with disabilities. Participating with able-bodied peers might be empowering, and important for building the long-term motivation needed for pursuing a pathway toward elite parasport [41]. The potential role of integrated sport environments in long-term development towards elite performance in athletes with a disability seems thus to be an important avenue for further research. Furthermore, these empirical findings lend to the premise that an age-related athlete development model may not be an optimal approach in parasports, but need rather to focus on allowed people with disabilities participating in activity together with peers and able-bodied friends.

### 4.2. Sport ‘Sampling’ in Childhood

Multiple studies have shown that elite able-bodied athletes have participated in several sports through childhood [22,29,42,43]. The principle of sport sampling in accordance with the DMPS framework is built on the concept that children experience several different sports with a focus on deliberate play and with a low focus on deliberate practice [44]. Sport sampling allows children to experience and develop different motor skills and cognitive skills that are required when specializing in one sport [1,45]. The elite-level para-athletes in the current sample did indeed reflect upon participating and trying out multiple sports throughout childhood, thus adhering to the notion of sport sampling as an important aspect of the developmental trajectory towards world-class performance.

A distinction emerges, however, between the transcripts in the current study and that of the DMSP, when considering the relative contribution of deliberate practice and deliberate play in sport sampling. The sport participation in childhood appeared to be highly coach-led, organized, and focused on sport-specific skills, which are typical indicators of deliberate practice [1,45]. Thus, albeit the athletes in the current study participated in various sports several times a week (a multi-sport approach), there seemed to be less opportunity for sport-related activities that can be less adult-organized and more self-regulated/peer-regulated, in which the athletes themselves are discovering and exploring activities in deliberate play [1,15,45].

Typically, deliberate play with an unorganized approach to skill acquisition is often designed to maximize enjoyment in contrast to deliberate practice where the specific purpose of the organized training sessions is to increase performance (e.g., not for enjoyment or external rewards) [45]. There are, however, examples of studies supporting that deliberate practice activities can result in enjoyable effects [46], and the athletes in the current study indeed reported to experience enjoyment when participating in coach-led and organized sport. Importantly, this sport participation facilitated social contact with their typically developing and able-bodied peers. This illustrates that some postulates generated from the DMPS are not directly transferable to developmental pathways towards elite-level parasport. Participating in organized sport activities with peers might lead athletes with a disability to discover sport possibilities and become a part of a group where teammates can contribute to the development of sport expertise by sharing experiences and knowledge about the sport and help match their training exercises towards their current skill level [47].

### 4.3. Youth Sport Transitions

The youth developmental period seemed to contain the greatest inter-individual differences between the athletes in the current study. Indeed, there are examples of athletes who have reached the high-performance levels without any specific organized training during adolescence, but rather have similarities with the developmental trajectory in the DMSP which contains a high amount of deliberate play and activities that focus on fitness and health [14]. This clearly demonstrates that any developmental history of sport is highly individualized, and that athletes report a mixture of the pathways indicated in the DMSP. Indeed, multiple studies [14,22,43,44,48] indicate that expert able-bodied athletes did not follow a particular trajectory in the youth sport period, but rather changed between different trajectories when developing toward expert performance.

The findings in the current sample of expert athletes with a physical disability are that they initiated their focus on a main sport relatively late in adolescence and are in line with other studies, where Huxley et al. [49] showed that the most para-athletes did not focus on their main sport in both training and competition before at least the age of 16. As captured by the DMPS model, para-athletes are not physically or psychosocially ready to invest in specialized training before the late adolescent period. While able-bodied athletes often engage considerably in their primary sport also during childhood [50], it seems as though athletes in the current sample had an extended sampling period before they followed a more specializing trajectory. As a consequence of these findings, it might be that para-athletes must be recommended to sample a variety of sport before specialization in late adolescence. Furthermore, these transitions in youth sport seem to be highly influenced by sometimes random encounters with, e.g., elite sport federations, teams and/or coaches that can assist in developing individualized training schedules tailored for athletes with disabilities. It thus appears that across sport for both able-bodied athletes and athletes with a disability, that in order to reach elite-level sport at the international level, young athletes need to initiate highly structured and organized training no later than during their mid-adolescence years [49,51,52]. Similarly, transitions also need to take place in terms of competition levels during youth sport. As a part of the specializing pathway, most of the athletes in the current study first entered international level competitions at this age, and four of the athletes also reported achieving their first international medal at a junior age (17–19).

The important role of individualized equipment is a recurring theme in sport for athletes with a disability and analyses of transcripts from the current study also clearly indicated that in order to transit to specialized and high-level practice and competition, participation often requires costly equipment that might be difficult to find altogether. In Kean et al. [21] investigation of wheelchair basketball players, financial barriers were also reported due to expensive equipment. These barriers have in some instances led the athletes in the current sample to buy their own equipment, which follows from the transitions towards developing strong intentions and motivations towards becoming elite-level performers. These motivations and intentions seem to emerge alongside the increased involvement in a particular organized sport and shift towards more organized and professional training and competition environments. The need for better equipment thus follows from these changes, and the transitional youth sport period might thus contain several biopsychosocial challenges to overcome if one is to pursue a pathway towards elite-level parasport.

### 4.4. Significant Others in a Sport Context

As mentioned previously, parents, peers and family are influential socializing agents [38] for early sport-related learning experiences for the current sample of elite athletes. Similarly, coaches, sport organizations and other athletes are deemed especially important in youth sport transitions towards the development of elite-level performance. It is beyond the scope of models such as the DMSP to pinpoint such key aspects of elite-level pathways, albeit it was a recuring theme amongst the individual retrospective reflections provided in the current study. Altogether, the significant others as jointly termed in the current study (e.g., parents, family, peers, friends, coaches, sport organizations, etc.) seemed to have created a sport environment around the young developing athletes with disabilities that shared two interlocked traits: (i) Focus on further development, not necessarily performance and (ii) seeing beyond the limitations that might reside with the disabilities. The various person’s influence on the athlete might have different contributions at different times, but need to focus on what can be done instead of what cannot be done in training and practice. It seems to be of vital importance that people are knowledgeable of the interaction between technical and tactical sport-specific skills and the different individual disabilities. Coaches, especially, need to go beyond the knowledge required for able-bodied practice to provide athletes with disability-relevant and sport-specific input [53,54]. Altogether, it seems highly important for athletes with a disability that influential persons in a sport context foster a motivational climate that focuses on skill improvement and individual progress.

### 4.5. Limitations and Future Directions

Several limitations exist with this study, that will motivate further examination. Typical for an exploratory and qualitative study, the focus was on a modest sample of athletes with a physical disability. The athletes had sustained performance at the highest levels, however, and such top-ranked athletes do not typically exist in high numbers. Hence, they were not just competing at the highest levels, they were also among the very best in the world in their specific sport. Still, the study obtained insights from a small group of participants, and their responses may have differed from a quantitative study gathering data from larger samples of athletes with a physical disability. Furthermore, although no systematic differences seemed to emerge in the current study’s thematic analysis between those with congenital and acquired disabilities, important insights might still be gained if developmental stories are collected from these specific sub-samples. The current study was also explicitly viewed through the lens of the developmental model of sport participation, and it seems clear that additional in-depth interviews should focus on specific developmental periods in order to further elucidate facilitating factors and barriers. Cultural differences might also have substantial impact upon the perceptions of disability, thus limiting the current study findings to European and industrial societies with high levels of welfare. As the understanding of elite sport development in parasport is further increased, it should be possible to develop specific models that can be addressed by various methods, e.g., developing a survey instrument based upon the qualitative research.

## 5. Conclusions

The study examined developmental pathways towards elite-level performance, through childhood and adolescence, amongst athletes with a physical disability that can be defined as top-performers in their sports. The retrospective interviews and thematic analysis were viewed through the lens of the developmental model of sport participation and explored their perspectives and reflections in terms of the proposed pathways in the DMSP. The findings of the present study offer support for some of the propositions in the model, although also addressing some of the problems with applying non-disability athlete development models towards understanding athletes with a disability. It appears that early sport-related encounters typically in a family environment have been important for the athletes in the current sample, shaping their further interest in sport, and beginning the journey of seeing past their disability. This is typically followed by childhood experiences with a substantial amount of sport sampling through participating in a number of different organized and coach-led sports. This sampling facilitates contact with their able-bodied peers and seems in some instances to work as a substitute for the substantial amount of peer-led unorganized activities typically found in the developmental histories of able-bodied high-level athletes. The youth sport period seems to be particular transitional, in which further adaptation of equipment follows from the transition to more organized and structured practice and higher levels of competition in their main sport. The intention of becoming a high-level athlete emerges in the youth period, and family, coaches, friends and athletes all seem to play a role in sustaining motivation towards improvement and further progress. These findings, and those of others, might be applied in creating developmental models tailored towards parasport, in which they can inform policymakers and organizations, on how to best tailor the parasport experience.

## Data Availability

Not applicable, as the study includes data protected by the Norwegian Personal Data Act and The General Data Protection Regulation from the EU (GDPR).
